# Identification of Myocardial Insulin Resistance by Using Liver Tests: A Simple Approach for Clinical Practice

**DOI:** 10.3390/ijms23158783

**Published:** 2022-08-07

**Authors:** José Raúl Herance, Queralt Martín-Saladich, Mayra Alejandra Velásquez, Cristina Hernandez, Carolina Aparicio, Clara Ramirez-Serra, Roser Ferrer, Marina Giralt-Arnaiz, Miguel Ángel González-Ballester, Juan M. Pericàs, Joan Castell-Conesa, Santiago Aguadé-Bruix, Rafael Simó

**Affiliations:** 1 Medical Molecular Imaging Research Group, Vall d’Hebron Research Institute (VHIR), Nuclear Medicine, Radiology and Cardiology Departments, Vall d’Hebron University Hospital, Autonomous University Barcelona, 08035 Barcelona, Spain; 2 CIBERBBN (Instituto de Salud Carlos III), 28029 Madrid, Spain; 3 BCN Medtech Group, Information and Communication Technologies Department, Pompeu Fabra University, 08018 Barcelona, Spain; 4 Diabetes and Metabolism Research Group, Vall d’Hebron Research Institute (VHIR), Endocrinology Department, Vall d’Hebron University Hospital, Autonomous University Barcelona, 08035 Barcelona, Spain; 5 CIBERDEM (Instituto de Salud Carlos III), 28029 Madrid, Spain; 6 Clinical Biochemistry Research Group, Vall d’Hebron Research Institute (VHIR), Biochemical Core Facilities, Vall d’Hebron University Hospital, Autonomous University Barcelona, 08035 Barcelona, Spain; 7 Liver Diseases Research Group, Vall d’Hebron Research Institute (VHIR), Liver Unit, Vall d’Hebron University Hospital, 08035 Barcelona, Spain; 8 CIBEREHD (Instituto de Salud Carlos III), 28029 Madrid, Spain

**Keywords:** type 2 diabetes, myocardial insulin resistance, non-alcoholic fatty liver disease, cardiovascular risk

## Abstract

Background: We report that myocardial insulin resistance (mIR) occurs in around 60% of patients with type 2 diabetes (T2D) and was associated with higher cardiovascular risk in comparison with patients with insulin-sensitive myocardium (mIS). These two phenotypes (mIR vs. mIS) can only be assessed using time-consuming and expensive methods. The aim of the present study is to search a simple and reliable surrogate to identify both phenotypes. Methods: Forty-seven patients with T2D underwent myocardial [18F]FDG PET/CT at baseline and after a hyperinsulinemic–euglycemic clamp (HEC) to determine mIR were prospectively recruited. Biochemical assessments were performed before and after the HEC. Baseline hepatic steatosis index and index of hepatic fibrosis (FIB-4) were calculated. Furthermore, liver stiffness measurement was performed using transient elastography. Results: The best model to predict the presence of mIR was the combination of transaminases, protein levels, FIB-4 score and HOMA (AUC = 0.95; sensibility: 0.81; specificity: 0.95). We observed significantly higher levels of fibrosis in patients with mIR than in those with mIS (*p* = 0.034). In addition, we found that patients with mIR presented a reduced glucose uptake by the liver in comparison with patients with mIS. Conclusions: The combination of HOMA, protein, transaminases and FIB-4 is a simple and reliable tool for identifying mIR in patients with T2D. This information will be useful to improve the stratification of cardiovascular risk in T2D.

## 1. Introduction

Type 2 diabetes (T2D) is a highly prevalent multifactorial disease caused by a combination of genetic and lifestyle factors which represents a huge societal problem associated with a significant economic burden for all healthcare systems [1,2]. T2D is characterized by high levels of plasma glucose caused mainly by pancreatic β-cell dysfunction and inefficient action of insulin, known as insulin resistance (IR), which occurs in cells of insulin-sensitive key organs and tissues such as the heart or skeletal muscle [1,3]. These high levels of plasma glucose cause mainly micro- and macrovascular complications [4,5]. Likewise, IR causes cells to not respond to the action of insulin to incorporate glucose and, thus, obtain energy by maintaining a normal metabolism [1,6]. The deficit of glucose in cells causes metabolic alterations and changes in oxidizable substrates to obtain energy, triggering severe pathological complications [7,8]. Therefore, both chronic hyperglycemia and IR have been related to the appearance of cardiovascular diseases (CVD) such as coronary artery diseases (CAD), stroke, and cardiomyopathies in T2D [6,8,9,10].

Myocardium is a striated muscle that constitutes the main tissue of the walls of the heart, and plays crucial roles for contraction, the basic function of the heart [11]. The main cells of the muscle are cardiomyocytes that are insulin-sensitive cells and, consequently, can be affected by the IR related to T2D. Many clinical trials have shown that systemic IR is an independent risk factor for heart failure and cardiovascular death [12]. In addition, increasing evidence points to IR as the primary etiologic factor in the development of non-ischemic heart failure [13,14].

A number of preclinical studies have shown that myocardial IR (mIR), which is characterized by inefficient energy metabolism, co-occurs with systemic insulin resistance and contributes to post-ischemic heart failure [15]. The presence of mIR should be put in the context of the general IR, in which the main contributors are the liver, muscles other than myocardium, and the adipose tissue. Some researchers have suggested not only that the heart is a target organ of systemic insulin resistance, but also that local mIR is a distinct risk factor for heart failure [16]. The myocardium normally responds to injury by altering substrate metabolism to increase energy efficiency. Insulin resistance prevents this adaptive response and can lead to further injury by contributing to lipotoxicity, sympathetic up-regulation, inflammation, oxidative stress, and fibrosis [13].

We have recently reported the presence of mIR in around 60% of patients with T2D patients without previous CVD [17]. These patients present higher myocardial radiodensity and a significantly higher coronary artery calcium score (CACs), a reliable biomarker of cardiovascular risk, when compared with those patients with insulin-sensitive myocardium (mIS). In addition, we observed that systemic IR is mainly accounted by patients with mIR, thus underlying the important role of the myocardium in glucose metabolism. This study was performed using 18F-fluorodeoxyglucose (18F-FDG) positron emission tomography (PET/CT) before and after a hyperinsulinemic–euglycemic clamp (HEC). This is an expensive, radioactive, time-consuming and cumbersome examination that cannot be proposed for daily clinical practice. Therefore, a more accessible, cheap and widely applicable surrogate to identify patients with mIR is needed.

In the present study, we examined a myriad of plasma biochemical parameters aimed at obtaining a reliable surrogate that permits us to differentiate the two phenotypes of T2D patients: mIR and mIS.

## 2. Results

The general results of PET/CT before and after HEC were previously reported [17]. In summary, the first (baseline) PET/CT showed in all patients a very low left ventricular [18]FDG uptake. After HEC, we were able to observe two clear behaviors: (1) A pronounced enhancement in [18]FDG uptake in 21 cases (44.7%), thus revealing an insulin-sensitive myocardium (mIS). (2) A marginal increase in [18]FDG uptake in the remaining 26 (55.30%) patients, thus indicating the presence of an insulin-resistant myocardium (mIR).

### 2.1. General Characteristics of T2D Patients

The main clinical and biochemical variables of T2D patients included in the study, taking into account the myocardial insulin resistance (mIS vs. mIR), are shown in Table 1. The antidiabetic treatment is displayed in Table 2.

### 2.2. Biomarkers Discriminating between mIR and mIS

Baseline and post-HEC biochemical variables were divided by mIS and mIR subgroups and analyzed. The variables significantly different between both groups at baseline are shown in Table 1. As can be seen, HOMA-IR, ALT, AST, GGT and protein were significantly higher in the mIR group than in the mIS group. However, after HEC, we found that only ALT and FFA were significantly increased in the mIR group: 29 IU/L [16:42] vs. 18 IU/L [16:21] (*p* = 0.0180), and 0.32 mmol/L [0.18:0.38] vs. 0.20 mmol/L *p* = 0.0149, respectively. We did not find any difference between males and females when comparing mIR vs. mIS and neither of the biochemical variables were useful for discriminating both phenotypes both at baseline and after HEC.

**Figure 1:**
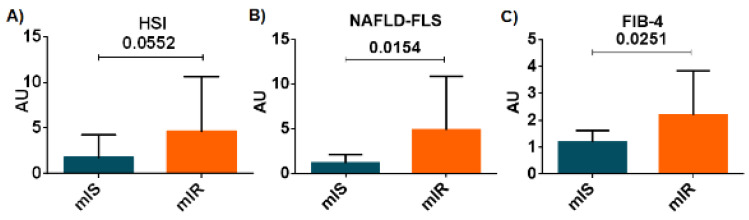
Differences between HIS (**A**), NAFLD-LFS (**B**) and FIB-4 (**C**) indexes between both phenotypes of T2D patients (ISm in blue and IRm in orange). Data are shown as mean ± SD.

Since liver enzymes were the most closely associated with mIR, we wanted to explore whether the hepatic steatosis index (HSI), a simple screening tool reflecting non-alcoholic fatty liver disease (NAFLD), was different between mIR and mIS patients [18]. We found a higher HIS in patients with mIR in comparison with those patients with mIS but the differences did not reach statistical significance (2.35 [1.06:5.62] vs. 1.16 [0.53:1.80]; *p* = 0.055) (Figure 1A). In addition, we examined whether the non-alcoholic fatty liver disease–liver fat score (NAFLD-LFS) [19], an index that integrates IR and hepatic damage, could be able to discriminate patients with and without mIR. We found that patients with mIR presented higher values than patients with mIS (2.58 [1.08:8.31] vs. 1.05 [0.47:1.76]; *p* = 0.0154) (Figure 1B). Finally, the usefulness of an index of hepatic fibrosis (FIB-4) was assessed [20]. We found a significantly higher FIB-4 score in patients with mIR in comparison with patients with mIS (1.65 [1.07:2.73] vs. 1.18 [0.82:1.49]; *p* = 0.025) (Figure 1C).

**Figure 2:**
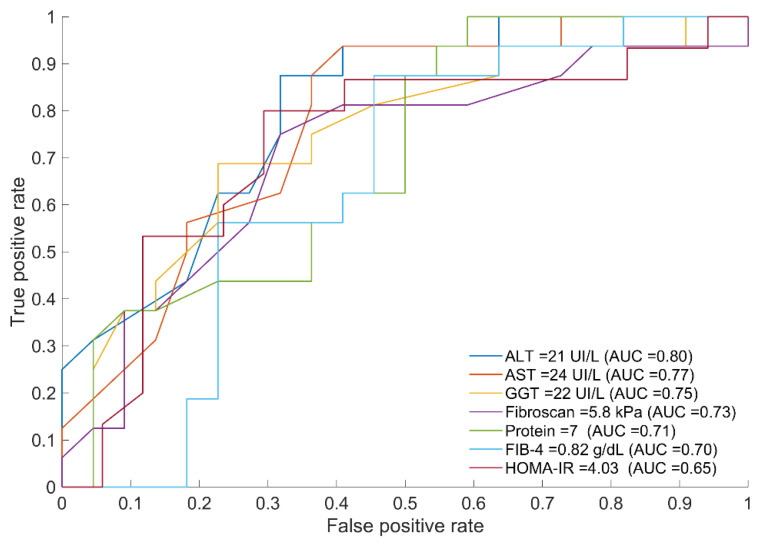
ROC curves of variables displaying the optimal threshold for splitting the two groups (mIR vs. mIS) together with their threshold and AUC values. The corresponding ROC metrics (sensitivity and specificity) are as follows: ALT (0.88, 0.68), AST (0.88, 0.64), GGT (0.69, 0.77), Protein (0.68, 0.75), FIB-4 (0.31, 0.95), and HOMA-IR (0.56, 0.77).

Since the baseline (fasting state) is the current state for performing biochemical analysis, thresholds and AUC for optimal discrimination at baseline between both groups (mIR and mIS) were obtained for the variables significantly different between groups (Figure 2).

**Figure 3:**
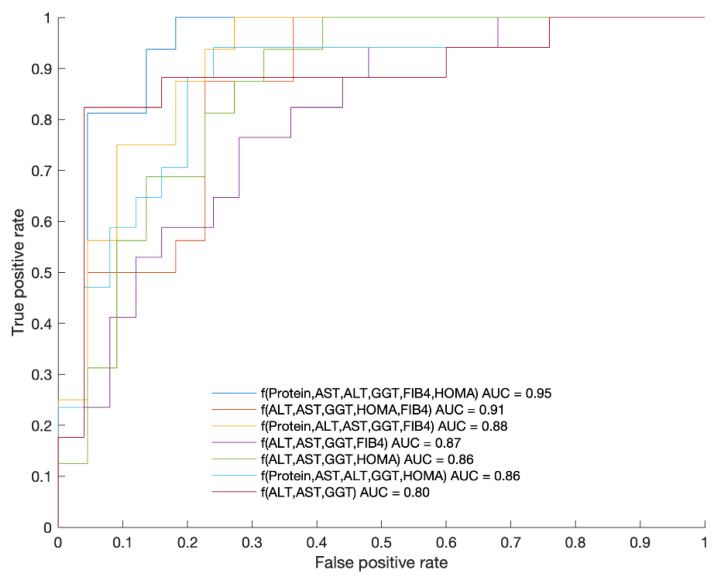
ROC curves of each of the presented functions as a combination of variables displaying the AUC values. The corresponding ROC metrics (sensitivity and specificity) are as follows: f(Protein, AST, ALT, GGT, FIB-4, HOMA-IR) (0.81,0.95), f(AL, AST, GGT, HOMA-IR, FIB-4) (1.00,0.73), f(Protein, ALT, AST, GGT, FIB-4) (0.82,0.96), f(ALT, AST, GGT, FIB-4) (0.88,0.80), f(ALT, AST, GGT, HOMA-IR) (0.88,0.77), f(Protein, AST, ALT, GGT, HOMA-IR) (0.69,0.86), f(ALT, AST, GGT) (0.53,0.88). The best function obtained for splitting both groups with AUC = 0.95 was: f = 27.444 + (−2.9636 × [protein]) + 0.25195 × [AST] + (−0.36118 × [ALT]) + 0.014958 × [GGT] + (−3.6456 × [FIB-4]) + (−0.057339 × [HOMA-IR]). The *p* value of this function was 0.000043 with a threshold to split both cohorts of 1.37.

In addition, different models combining the variables displayed in Figure 2 were analyzed to dichotomize both phenotypes (mIR vs. mIS). The results are shown in Figure 3.

The best model was the combination of Protein, AST, ALT, GGT, FIB and HOMA (AUC = 0.95; sensibility: 0.81; and specificity: 0.95). Since HOMA is not always available in the daily clinical practice and it cannot be used in those patients receiving treatment with insulin, we wanted to examine the result obtained by leaving HOMA out of the model, and the result was AUC = 0.87; sensibility: 0.82; and specificity: 0.96.

### 2.3. Hepatic Fibrosis Measured by Fibroscan

**Figure 4:**
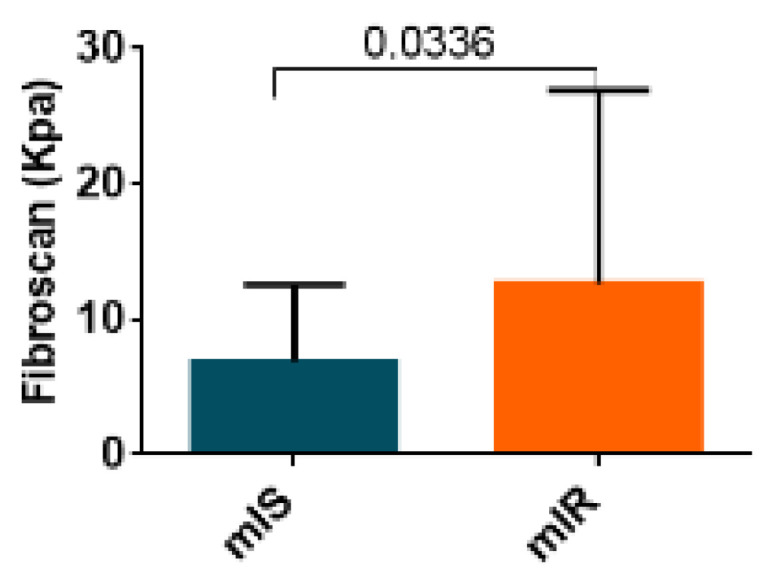
Results of liver assessment of fibrosis by fibroscan of both groups patients with T2D (mIR and mIS). Data are shown as median ± SD.

Since FIB-4 is a reliable index of hepatic fibrosis, we wanted to examine whether the degree of hepatic fibrosis measured by fibroscan was related to the presence of mIR. We observed significantly higher levels of fibrosis in patients with mIR than in those with mIS (9.10 [5.43:16.08] vs. 5.35 [3.70:6.38]; *p* = 0.034) Figure 4. ROC analysis of fibroscan data showed an AUC = 0.73 with a sensibility of 0.80 and specificity of 0.71.

**Figure 5:**
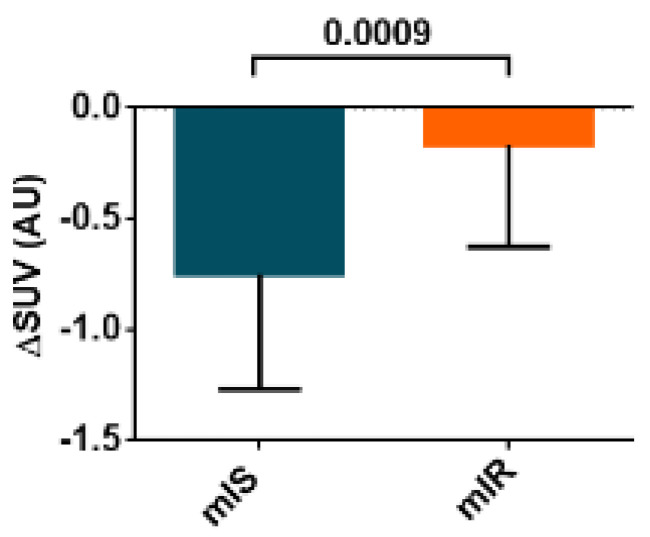
ΔSUV in liver after performing an HEC procedure of both groups (mIR and mIS). Data are shown as median ± SD.

In addition, patients with mIR presented a reduced glucose uptake by the liver in comparison with patients with mIS (Figure 5).

## 3. Discussion

Myocardial insulin resistance (mIR) has been hypothesized as a risk factor of CVD and cardiomyopathies in T2D patients [17,21]. Approaches based on dynamic PET/CT under HEC conditions have linked the myocardial [18F]FDG utilization rate to mIR [18,19]. Our group clearly identified T2D patients with mIR for the first time in a proof of concept using two [18F]FDG PET/CTs per patient (baseline and post-HEC) [17]. Since this methodology can hardly be proposed for current clinical practice, cost-effective and reliable surrogates are needed. In the present study, we provide evidence that transaminases and FIB-4 (an index of hepatic fibrosis) are the best predictors of mIR in patients with T2D without previous history of cardiovascular events.

We found that the best model to predict the presence of mIR was the combination of protein, AST, ALT, GGT, FIB-4 and HOMA-IR at baseline (AUC = 0.95; sensibility: 0.81; specificity: 0.95). Since HOMA-IR is not always available in the daily clinical practice and it cannot be used in those patients receiving treatment with insulin, we examined the result obtained by leaving HOMA-IR out of the model, and the result was AUC = 0.87 with a sensitivity of 0.82 and a specificity of 0.96. This means that general practitioners could have a useful index to estimate the presence of mIR in patients with T2D.

Regarding the variables altered after the HEC procedure, the higher serum levels of FFA and ALT were found in the IRm subgroup. The increase in FFA in those patients with mIR could be expected because it is well-established that FFAs decrease myocardial [18F]FDG uptake [22]. It is well-established that the primary fuel for the myocardium is represented by FFAs in the fasting state and glucose in the fed state [23,24]. Myocardial glucose uptake is inversely associated with serum FFAs in both healthy and insulin-resistant conditions, and such a relationship explains the bulk of the individual variation in myocardial metabolism [24,25]. However, the fact that levels of FFA were significantly different between mIR and mIS patients only after HEC but not at the baseline/fasting state is intriguing and merits further investigation. The higher plasma levels of ALT after HEC in diabetic patients with mIR in comparison with patients with mIS have not previously reported and speak in favor of the robustness of ALT in dichotomizing both groups (mIR and mIS). Nevertheless, for the practical point of view, the fasting/baseline results are the only useful ones for clinical purposes.

Overall, our results confirm previous findings suggesting the existence of a liver–heart axis [26,27,28,29]. To the best of our knowledge, this is the first report linking hepatic liver disease and mIR for T2D without previous CVD. In addition, we demonstrated that patients with mIR presented a significantly lower glucose uptake by the liver than patients with mIS. This result reveals the existence of an association between the myocardium and liver IR, which is essential to understand our findings. The liver is a central hub for lipid metabolism and endogenous glucose production; therefore, the liver is crucial for systemic glucose and lipid homeostasis. Non-alcoholic fatty liver disease (NAFLD) has become the most prevalent chronic liver disease, affecting approximately one-quarter of adults worldwide. NAFLD encompasses a pathological condition characterized by the ectopic deposition of adipose tissue in the liver, in the absence of secondary causes for hepatic fat accumulation, such as excessive alcohol intake, steatogenic drugs, and hereditary disorders [30]. Beyond liver-related morbidity and mortality, there is increasing evidence that patients with NAFLD are also at high risk of cardiovascular diseases (CVDs) [31]. Notably, 25% to 40% of patients with NAFLD also have CVD, and CVD accounts for a higher proportion of mortality than liver-related death in patients with NAFLD [32]. Recently, experts reached a consensus that NAFLD does not reflect the current knowledge, and metabolic-dysfunction-associated fatty liver disease (MAFLD) was suggested as a more appropriate term [33]. Although NAFLD or MAFLD is among the major forces driving CVD, the mechanistic relationship with CVD remains to be elucidated. Possible mechanisms include, but are not limited to, insulin resistance, low-grade systemic inflammation, oxidative stress and neuroendocrine activation [29]. Besides these common mechanisms, changes in protein secretion from the fatty liver also contribute to the pathogenesis of CVDs. In fact, the liver has recently been recognized as an endocrine organ that secretes hepatokines, which are proteins secreted by hepatocytes that can influence metabolic processes through autocrine, paracrine, and endocrine [34,35,36]. In the setting of MAFLD, several hepatokines can be downregulated such as sex hormone-binding globulin (SHBG), angiopoietin-like protein 4 (ANGPTL4) and adropin, whereas others such as fetuin-A, fetuin-B, hepassocin, leukocyte cell-derived chemotaxin 2 (LECT2), retinol-binding protein 4 (RBP4), selenoprotein P (SeP) and fibroblast growth factor 21 are upregulated [29,35]. The specific contribution of hepatokine imbalance on the uptake of glucose by the myocardium is beyond the scope of this paper but will be a piece of cutting-edge research in the coming years.

Lautamäki et al. demonstrated for the first time that liver fat content is an independent, significant mediator of myocardial glucose uptake in T2D patients with CAD [37]. In addition, they suggested a direct relationship between NAFLD and coronary atherosclerosis by showing impaired coronary flow reserve and an increment in markers of low-grade inflammation in patients with NAFLD. Our findings reinforce and extend this concept to patients with T2D without CVD. In addition, our findings suggest that, rather than steatosis, it is the presence of liver fibrosis which is the most important factor contributing to mIR. In fact, we found significantly higher levels of fibrosis in patients with mIR than in those with mIS. This result suggests that pro-fibrotic molecules might be essential players in linking MAFLD and mIR. Liver fibrosis has been suggested to be an independent predictor of the incidence of CVD and CV events in patients with NAFLD [38,39,40] and has a crucial role in the development of CVD through coronary microvascular dysfunction [41]. Recently, Liu et al. showed that liver fibrosis score metrics were significantly associated with the occurrence of recurrent cardiovascular events in patients with coronary artery disease and prior cardiovascular events [42]. It should be noted that hepatic lipid accumulation may not be toxic, and can also function as a protective mechanism to buffer the lipotoxic effects of FFA to hepatocytes. The progression of pure hepatic steatosis to liver fibrosis remains silent and underdiagnosed until its development into cirrhosis, characterized by severe liver fibrosis. In our cohort, we found a significantly higher proportion of fibrosis (≥6 Kpa) in patients with mIR than mIS (70% vs. 18.7%). Hepatic steatosis and incipient liver fibrosis may be reversible at early stages, and, in this regard, the accurate quantification of these hepatic phenotypes becomes central to the design of preventive strategies involving lifestyle modifications and therapeutic interventions [43,44]. It should be underlined that the level of transaminases in those patients with mIR was in the normal range. Furthermore, a high percentage of patients with T2D with liver fibrosis also presented transaminases in the normal range, thus making difficult the clinical suspicion. These findings are in agreement with previous reports [45,46,47] and suggest being proactive in identifying T2D at risk of having mIR by using the mentioned algorithms.

Finally, the observed relationship between glucose uptake in the myocardium and the liver point mIR is a key factor in accounting for the increased CV risk in patients with MAFLD. The pathophysiological interactions between the liver and heart can be classified into three groups: (1) liver disease resulting from heart disease; (2) heart disease resulting from liver disease; (3) systemic conditions affecting the heart and the liver at the same time. Our findings suggest that T2D could be one of these systemic diseases altering, simultaneously, glucose uptake in both the liver and the heart. Nevertheless, as mentioned above, a primary pathogenic role of hepatokine mediator/s should be underlined.

There are some limitations in this study. First, we did not include a healthy control group, and the number of patients with T2D who were enrolled was low. The main reason is because PET/CT combined with HEC is cumbersome and time-consuming. In addition, using two PET/CTs on the same patient is limited due to ethical issues. Second, we did not confirm the presence and degree of hepatic fibrosis with additional histological assessments. Third, the withdrawal of the antidiabetic drugs one day before PET/CT could not be sufficient to assure a correct wash out. However, the insulin-mediated glucose uptake by using HEC overrides any eventual marginal action of insulin sensitizers or GLP-1 in terms of increasing the myocardial glucose uptake. In this regard, it should be noted that we observed a very low left-ventricular [18]FDG uptake in all patients in the first (baseline) PET/CT, and we did not observe any influence on [18]FDG uptake from antidiabetic drugs at both baseline and post [18F]FDG PET/CT. Fourth, although BMI was similar in mIR and mIR patients, a specific study on body composition was not performed and, therefore, a potential link between adipose tissue and mIR cannot be ruled out. Finally, studies on the long-term clinical consequences of mIR are needed.

In summary, our results add valuable information to understanding the “crosstalk” between the liver and the cardiovascular system. We provide evidence that T2D individuals with mIR can be identified by using a simple algorithm based on the plasma levels of proteins and transaminases. This information will be useful to improve the stratification of cardiovascular risk in patients with T2D, and will aid the development of new therapeutic strategies aimed at abrogating the underlying vicious circuits between the liver and the heart.

## 4. Material and Methods

### 4.1. Subjects

This proof-of-concept observational study comprised forty-seven T2D patients and was conducted according to the tenets of the Helsinki Declaration after approval by the Ethic Committee of the Vall d’Hebron University Hospital (ClinicalTrials.gov: NCT02248311). Written informed consent of all participants was obtained.

The inclusion criteria were: T2D patients from 50 to 79 years old, with at least 5 years from the diagnosis. The exclusion criteria were: (1) type 1 diabetes, (2) any previous CVD event, (3) patients with known cardiac pathology, (4) any contraindication or claustrophobia for the PET/CT, (5) any pathology related to a short life expectancy, (6) daily alcohol drinker, (7) smokers who did not stop smoking at least 1 year before (non-smoker and ex-smoker were recorded), (8) treatment with any cardiotoxic medication.

Subjects were recruited at the Endocrinology Department of Vall d’Hebron University Hospital. Among a total of 250 clinical records of T2D patients reviewed, 72 met the inclusion criteria, but only 51 agreed to participate in the clinical trial. Four patients dropped out of the study before its completion.

### 4.2. Study Design

**Figure 6:**
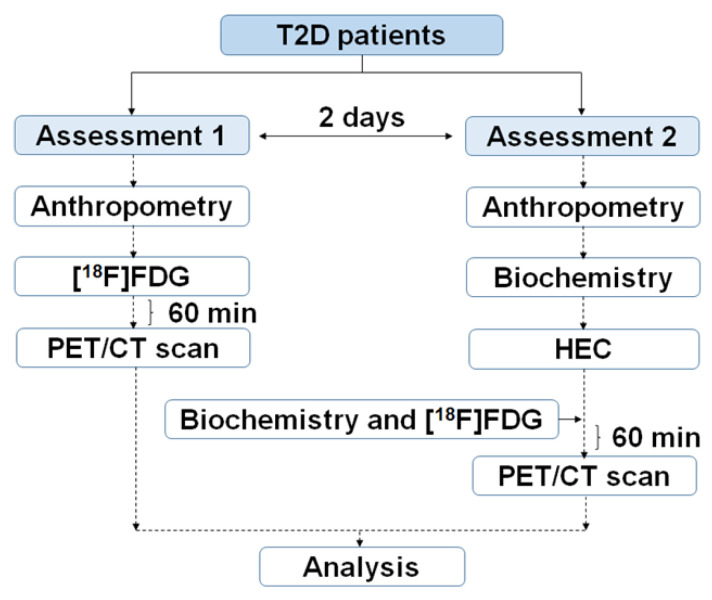
Outline of the clinical trial to obtain PET/CT, biochemical and anthropometrical data in T2D patients.

Two [18F]FDG PET/CT studies per patient were implemented: one at baseline and another after performing a HEC. The HEC procedure was performed as previously reported with minor modifications [48,49]. The two [18F]FDG PET/CT scans were performed for each patient in a random order within two days under at least 8 h of fasting conditions and after the withdrawal of any medication the day before. A dose of 1.9 MBq/Kg of [18]FDG was IV-administered to patients before each scan session. PET/CT acquisitions protocols and data processing and analysis were described by our group previously [17]. Several anthropometric measurements and blood samples for biochemical analysis were obtained during the procedure following the flow chart shown in Figure 6.

### 4.3. Biochemical Analysis

Peripheral blood samples were collected under fasting conditions. Plasma samples for 1H-RMN analysis were stored at −80 °C at Vall d’Hebron Biobank. Biochemical analysis was performed at the Biochemistry Core Facilities of Vall d’Hebron University Hospital using standardized and validated routine methodologies. The following parameters were determined: blood count, glucose, HbA1c, fructosamine (FA), urea, creatinine (Cr), glomerular filtration (GF), sodium (Na), potassium (K), phosphate (PH), calcium (Ca), aspartate aminotransferase (AST), alanine aminotransferase (ALT), gamma glutamiltransferase (GGT), alkaline phosphatase (ALP), total cholesterol (C), high-density lipoprotein (HDL) cholesterol (C-HDL), low-density lipoprotein cholesterol (C-LDL), triglyceride (TG), total free fatty acids (FFA), protein (P), albumin, C-peptide, troponin (TN), chloride (Cl), total bilirubin (BR) and esterified bilirubin (E-BR). Non-routine laboratory parameters such as insulin, leptin and IL4 were also determined. Insulin was assessed by chemiluminescence immunoassay (Atellica IM, Siemens Healthineers, Erlangen, Germany), leptin analysis was performed by ELISA (Diagnostics Biochem Canada Inc., London, ON, Canada), and IL6 by electrochemiluminescence immunoassay (Cobas e2801, Roche Diagnostics, Basel, Switzerland) following strictly the instructions provided by the manufacturer.

The fibrosis index based on four factors (FIB-4) was determined by the standard equations using age, ALT, AST and platelet counting. 1H-RMN analysis was performed following the previous methodology described by our group to determine VLDL (CH) and VLDL (aliphatic chain) [50].

### 4.4. Fibroscan

Liver stiffness measurement (LSM) was performed using transient elastography (Fibroscan 502 Touch, Echosens, Paris, France). According to the usual standard procedure, measurements were performed under fasting conditions with at least 10 valid measurements and an interquartile to median ratio ≤ 30% [51]. Data are expressed in kilopascals (kPa). Normal LSM values varied between 4 and 6 kPa and values ≥ 6 kPa were considered abnormal and suggestive of liver mild fibrosis/disease.

### 4.5. Statistical Analysis

Data are expressed as median and interquartile range (IQR). Data normality was assessed with the Shapiro–Wilk test. The Kolmogorov–Smirnov test was used for group comparisons, as appropriate. Spearman’s correlation analysis and ROC curves were performed for variable associations. An ROC analysis was also performed to determine the utility of variables to dichotomize groups and obtain thresholds for fingerprints. With the extracted altered parameters for T2D phenotype segregation, new multi-variable functions were computed using the generalized linear model approach with specifications for binomial distribution and logistic-type canonical link function. This was carried out to compute alternative variable definitions for patient classification based on a combination of the mentioned parameters. ROC curves using the newly defined variables were obtained to assess the best combination according to the highest AUC value. Thresholds for each of the included parameters as well as for the combination of them were obtained by means of the same procedure as in the independent-variable assessment. All data were analyzed in GraphPad Prism (Version 6.01, San Diego, CA, USA) or R commander (Version 2.3-1, Hamilton, ON, Canada). *p* values below 0.05 were considered statistically significant.

## Figures and Tables

**Table 1:** Relevant biochemical features of T2D patients indicated as median ± interquartile range (IQR), except for age which is median ± SD.

	mIS	mIR	*p*
Age (years)	69 ± 2	66 ± 1	0.1837
Gender (M:F)	11:10 (21)	13:13 (26)	>0.9999
Glucose (mg/dL)	118 [109:142]	122 [114:147]	0.8893
BMI	30.46 [27.83:37.22]	31.16 [29.27:34.87]	0.5010
Cholesterol (C) (mg/dL)	161 [141:171]	176 [16:207]	0.1757
C-HDL (mg/dL)	48 [38:52]	43 [38:50]	0.3097
C-LDL (mg/dL)	84 [76:102]	105 [82:122]	0.3012
HbA1c (%)	7.05 [6.48:7.40]	7.40 [6.80:7.70]	0.3537
HOMA-IR	3.88 [3.21:5.26]	5.51 [4.39:9.98]	0.0335
ALT (IU/L)	21 [18:23]	30 [22:41]	0.0054
AST (IU/L)	17 [15:21]	29 [18:39]	0.0084
GGT (IU/L)	19 [14:27]	29 [23:48]	0.0285
Triglycerides (mg/dL)	109 [73:166]	124 [100:193]	0.2537
FFA (mmol/L)	0.62 [0.58:0.77]	0.75 [0.60:0.85]	0.2292
Protein (g/dL)	6.80 [7.00:6.80]	7.20 [6.93:7.48]	0.0273
Albumin (g/dL)	4.10 [3.90:4.20]	4.35 [4.10:4.50]	0.1814

**Table 2:** Antidiabetic treatment of patients included in the study.

	mIS	mIR
Metformin, n (%)	4 (23.53)	3 (11.54)
Metformin and/or other OA *, n (%)	3 (17.65)	9 (34.60%)
OA + GLP1-Ras, n (%)	2 (11.76)	2 (7.69%)
Insulin, n (%)	1 (5.88)	-
Insulin + OA, n (%)	3 (17.65)	8 (30.76)
Insulin + GLP1-RAs, n (%)	1 (5.88)	-
Insulin + OA + GLP1-RAs, n (%)	3 (17.65)	4 (15.38)

OA: oral antidiabetic agents; GLP1-RAs: GLP-1 receptor agonists. *: DPP-4 inhibitors, SGLT2 inhibitors, repaglinide.

## Data Availability

The datasets used and/or analyzed during the current study are available from the corresponding author on reasonable request.
